# Quantification of the UK five-point breast imaging classification and mapping to BI-RADS: to facilitate comparison of reporting, research and published literature

**DOI:** 10.1186/bcr2986

**Published:** 2011-11-04

**Authors:** K Taylor, PD Britton, S O'Keeffe, MG Wallis

**Affiliations:** 1 Cambridge University Hospitals NHS Foundation Trust, Cambridge, UK

## Introduction

The Royal College of Radiologists Breast Group has formalised the UK five-point breast imaging scoring system to encourage uniformity of reporting. The Breast Imaging and Reporting Data System (BI-RADS) is widely used throughout North America and Europe, and, unlike the UK scoring system, each BI-RADS category has an associated cancer likelihood. This study aims to quantify the cancer likelihood of each of the UK categories and map them to comparable BI-RADS categories to facilitate comparison of reporting, research and literature.

## Methods

Between January 2001 and December 2008, mammograms and ultrasounds performed in a symptomatic setting were prospectively UK scored and the percentage of cancer outcomes within each group calculated. These were then compared with the percentage incidence of the BI-RADS categories.

## Results

Of 23,741 separate assessment episodes, 15,288 mammograms and 10,642 ultrasound examinations were evaluated. There was direct correlation between UK scoring and BI-RADS for categories 1 and 5. UK score 2 lipomas and simple cysts correlated with BI-RADS 2, with the remaining UK score 2 lesions (mostly fibroadenomas) assigned to BI-RADS 3. BI-RADS 4 incorporates a wide range of cancer risk (2 to 95%) with subdivisions a, b and c indicating increasing, but unspecified, likelihood of malignancy. UK score 3 correlated with BI-RADS 4 a/b and UK score 4 corresponded with BI-RADS 4c.

## Conclusion

The cancer likelihood of the UK scoring has been quantified and mapped to the appropriate BI-RADS categories with equivalent cancer risks. This facilitates the sharing of UK research data and clinical practice on an international scale.

